# [Retracted] MicroRNA‑584 prohibits hepatocellular carcinoma cell proliferation and invasion by directly targeting BDNF

**DOI:** 10.3892/mmr.2024.13248

**Published:** 2024-05-20

**Authors:** Yanan Song, Guoyu Wang, Juhua Zhuang, Jing Ni, Suiliang Zhang, Ying Ye, Wei Xia

Mol Med Rep 20: 1994–2001, 2019; DOI: 10.3892/mmr.2019.10424

Following the publication of this paper, it was drawn to the Editor's attention by a concerned reader that the Transwell invasion assay data shown in Fig. 2C and D on p. 1997 were strikingly similar to data appearing in different form in other articles written by different authors at different research institutes that had either already been published, or were submitted for publication at around the same time (and in some cases, have subsequently been retracted).

Owing to the fact that the contentious data in the above article had already been published prior to its submission to *Molecular Medicine Reports*, the Editor has decided that this paper should be retracted from the Journal. The authors were asked for an explanation to account for these concerns, but the Editorial Office did not receive a reply. The Editor apologizes to the readership for any inconvenience caused.

